# High Prevalence of Chronic Viral Hepatitis and Liver Fibrosis among Mongols in Southern California

**DOI:** 10.1007/s10620-020-06499-6

**Published:** 2020-08-08

**Authors:** Tse-Ling Fong, Brian T. Lee, Mimi Chang, Khishigsuren Nasanbayar, Enkhjargal Tsogtoo, Delgerbat Boldbaatar, Esugen D. Dashdorj, Namuun E. Clifford, Arghun N. Dashdorj, Bo Ram Bang, Takeshi Chida, Carolina Lim, Masaya Sugiyama, Masashi Mizokami, Naranjargal J. Dashdorj, Ping Liu, Jeffrey S. Glenn, Naranbaatar D. Dashdorj, Takeshi Saito

**Affiliations:** 1 Division of Gastrointestinal and Liver Diseases, Keck School of Medicine, University of Southern California, Los Angeles California, USA; 2 Asian Pacific Liver Center, St. Vincent Medical Center, Los Angeles, California, USA; 3 Onom Foundation, Ulaanbaatar, Mongolia; 4 The Liver Center, Ulaanbaatar, Mongolia; 5 Genome Medical Sciences Project, National Center for Global Health and Medicine, Ichikawa, Chiba, Japan; 6 Division of Gastroenterology and Hepatology, Department of Medicine, Stanford University School of Medicine, Stanford, CA, USA

**Keywords:** hepatitis B, hepatitis C, hepatitis delta, cirrhosis, fatty liver

## Abstract

**Background:**

Mongolia is a highly endemic region for chronic hepatitis B (HBV), hepatitis delta (HDV), and hepatitis C (HCV) infections. Aim of this study was to comprehensively characterize chronic viral hepatitis among Mongols living in Southern California.

**Methods:**

Three screening events were conducted between August to November 2018, with 528 adult Mongols tested for HBV and HCV. HBsAg (+) individuals (CHB) underwent additional testing for HDV RNA and anti-HDV. Liver tests, platelet count and FibroScan™ were performed on CHB and chronic HCV (CHC) individuals.

**Results:**

51/534 were HBsAg reactive (9.7%) and all were foreign born. Mean age of CHB individuals was 37.8 (range 18–69) years. 46/51 were HBeAg (−). HBV genotypes were exclusively D2 or A1. 21/51 (41.2%) were anti-HDV (+) and 17/51 (33.3%) were HDV-RNA (+). HDV RNA (+) individuals had significantly higher ALT, Fibrosis-4 score and liver stiffness compared to HDV RNA (−) individuals. Incidence of advanced fibrosis was higher in HDV RNA (+) individuals (57% vs. 13%, p=0.013). 48 (9.1%) individuals were anti-HCV (+) and 19 (3.6%) were HCV RNA (+). Mean age of CHC individuals was 40.2 (range 28–71) years. Prevalence of anti-HCV (+) was higher among those born between 1945–1965 versus those born after 1965 (18.8% vs 7.9%, p=0.025). Genotype 1b was predominant. Incidence of cirrhosis was 7% among all participants.

**Conclusions:**

Mongols living in the US are at high risk for CHB and CHC infections. One-third of CHB individuals had CHD super-infection with advanced fibrosis. Universal screening for viral hepatitis in Mongols in the U.S. is mandatory.

## INTRODUCTION

Chronic hepatitis B (CHB) affects more than 257 million people world-wide with two-thirds of CHB infected people living in Asia and Sub-Saharan Africa (1). Although the overall prevalence of CHB is high in Asia, the prevalence in different countries varies; ranging from less than 0.5% in Japan to greater than 12% in Vietnam and Cambodia (1). Hepatitis delta virus (HDV) is a satellite virus that can infect individuals with hepatitis B virus (HBV) and 15–20 million people are estimated to be chronically infected with HBV and HDV (2). Chronic HBV/HDV infection is associated with a more rapid progression to cirrhosis and a higher incidence of hepatocellular carcinoma compared to patients with CHB mono-infection (2). While the prevalence of HDV is decreasing, it remains high in Mediterranean and Sub-Saharan countries and pockets of South America (2). Outside of Mongolia and pockets in Vietnam, chronic HDV infection in Asia is low (3 and 4).

It is estimated that there are 150 to 180 million individuals infected with hepatitis C virus (HCV) worldwide with a global prevalence rate of 2–3% (5). HCV prevalence is highly variable among different regions and individual countries; it is highest in countries in the Middle East and North Africa (> 2%) and lower in North America, Australia, Northern and Western Europe (<2%) (5).

The estimated number of CHB individuals in the U.S. ranges from 550,000 to 2 million of whom about 60% are foreign born (24). Although 40% of immigrants to the U.S. originate from Asia (7), they account for the majority of the 60% of foreign-born individuals in the U.S. with CHB. Mongolia is a central Asian country with a population of approximately 3 million people where chronic viral hepatitis B, D and C infections are highly endemic (3 and 7). Prevalence rates of CHB and chronic hepatitis C (CHC) among Mongols are 11.1% and 8.5% respectively (3), and up to 57% of Mongols with CHB are super-infected with HDV (7).

An estimated 30,000 Mongols live in the United States (U.S.) with the largest population residing in California. A previous study with sero-survey of Mongolian immigrants living in the Washington D.C area found CHB and CHC in 6.2% and 9.9% among tested individuals but testing for HDV was not performed (8). Asian-Pacific Liver Center (APLC) at St. Vincent Medical Center is a not-for-profit organization that provides education, medical care and regular screening of hepatitis B among various Asian communities in Southern California (9). Since 2007, APLC has screened more than 27,000 individuals in 321 screening events with more than 1400 cases of CHB identified. In addition to gaining a better understanding of the epidemiology of CHB in various Asian-American communities, these outreach programs provide linkage of care to participants. The aim of this study was to evaluate the prevalence of viral hepatitis among Mongols living in Southern California and comprehensively characterize the clinical features of chronic viral hepatitis diagnosed at community screenings.

## SUBJECTS AND METHODS

### SUBJECTS ENROLLMENT

This was a cross-sectional observation study. Between August and November 2018, 3 screening events for HBV and HCV that targeted Mongols were conducted at the Asian Pacific Liver Center (APLC) in Los Angeles in collaboration with the Onom Foundation, a non-governmental organization which initiated the Hepatitis Prevention, Control and Elimination Program in Mongolia but also extends its mission and services to overseas Mongols (10). These free screening events were advertised through social media and the Onom Foundation website (www.onomfoundation.org).

At the time of the screening, in addition to signing an informed consent, all participants completed a survey (in English or Mongolian) detailing their demographics (including country of birth, years in the U.S, family history of hepatitis and liver cancer). Prior to testing, all participants attended a lecture given in Mongolian language describing the significance of CHB and CHC infections and the rationale for HBV and HCV testing in the Mongolian community. An explanation regarding the possible HBV and HCV test results was also provided. All participants were notified of their HBV and HCV status by mail within 2 weeks which reiterated a detailed explanation of their test results in Mongolian and English. The notification letter to individuals who tested positive for hepatitis B surface antigen (HBsAg) and/or hepatitis C antibody (anti-HCV) specifically emphasized the need for medical follow-up and the option to have follow-up care, irrespective of insurance status and ability to pay, at the APLC. Additionally, these individuals were contacted by telephone and/or email for further evaluation with elastography and measurement of body mass index (BMI). Obesity was defined as 25.0 kg/m2, which is the cutoff for Asians, as suggested by the World Health Organization (11).

### BLOOD SCREENING and FIBROSCAN

Blood samples were obtained by venipuncture and tested for hepatitis B surface antigen (HBsAg) (Elecsys® HBsAg II, Roche Diagnostics), hepatitis B surface antibody and hepatitis C antibody (anti-HCV) (Elecsys® Anti-HCV II assay, Roche Diagnostics). All CHB individuals underwent additional testing for hepatitis B envelope antigen (HBeAg) (DiaSorin Liaison, Stillwater, MN, USA), anti-hepatitis B envelope antibody (anti-HBe) (DiaSorin), hepatitis B viral DNA (AmpliPrep/COBAS® TaqMan® HBV Test, v2.0, Roche Diagnostics).

All blood samples with quantifiable HBV DNA were subjected to HBV-genotyping via nested-PCR approach with primer sets targeting S gene (255bp, nucleotide positions 458–712). The amplicons of nested PCR were sequenced directly using the Sanger sequencing method as described previously (12). Briefly, nested PCR analyses were performed with two primer sets for 35 cycles (95°C, 15s; 58°C, 30s; 72°C, 30s) in 1st and 2nd PCR using Veriti thermal cycler (Applied Biosystems, Foster City, CA). The PCR products were sequenced with the inner primers used for 2nd PCR. Each of the sequence datasets was aligned by Clustal W in MEGA 7.0 and analyzed by neighbor-joining (N-J) in MEGA 7.0 with 1000 bootstrap replicates (13). Reference sequences to determine HBV genotype were retrieved from Hepatitis Virus Database (14).

In addition, all blood samples with positive HBsAg were tested for anti-HDV IgG Intensity Unit delta using Q-MAC assay as described previously (8) and the quantification of serum HDV RNA titer with in house qRT-PCR assay in the following method. The serum were first subjected to viral genome extraction using Zymo Quick-DNA/RNA Viral Kits (Zymo) followed by 1-step probe-based RT-qPCT analysis using the following primer set and probe: Forward: 5′-GGCWCTCCCTTAGCCATCCG-3′, Reverse: 5′-GGTCGGCATGGCATCTCCA-3′ and the probe: 5’-/56-FAM/CTCCTWCGGATGCCCAGGTCGGAC/36-TAMSp(TAMRA)/-3’.

All anti-HCV positive individuals were tested for hepatitis C virus RNA (COBAS® TaqMan® HCV Test v2.0) and hepatitis C virus genotype (Versant HCV genotype assay (LiPA) 2.0, LIPA). Liver tests, platelet count, and vibration controlled transient elastography (FibroScan®, Echosens™ Paris France) with controlled attenuation parameter (CAP) were performed on CHB and CHC individuals. All aspects of this study were approved by the Institutional Review Boards at St. Vincent Medical Center and Keck School of Medicine, University of Southern California.

### STATISTICAL ANALYSIS

Baseline characteristics and laboratory values were described as means (standard deviation), medians (interquartile range), or frequencies (percentages). Laboratory values were compared by HDV status. Student’s t-test or Mann-Whitney U test were used to compare differences as appropriate. Chi-square test was used to compare proportions as appropriate. All statistical analyses were performed using R 3.6.1 (R Foundation for Statistical Computing, Vienna, Austria).

## RESULTS

### Prevalence and Clinical Characteristics of HBV and HDV Infection

A total of 534 Mongols was screened. The mean age of the participants was 38 (range 4–69) years old, 55% were males and all but 3 individuals were born in Mongolia. The mean duration of living in the U.S. was 5 years. Fifty-three individuals were HBsAg reactive (9.9 %) and all but 2 were foreign born and living in the U.S. for mean of 6.4 (range 0.5–16) years. Prevalence of CHB was uniform across the age spectrum (Table 1). The mean age of CHB individuals was 38 (range 4–69) years and 58% were male. Family history of hepatocellular carcinoma was reported by 5 individuals. BMI and elastography with controlled attenuation parameter were measured in 21 and 34 individuals, respectively. The mean BMI among CHB individuals was 26.2 (range 15.5–33.2) kg/m^2^ and 62% were obese (defined by BMI ≥ 25 kg/m^2^) (11). Significant steatosis by CAP (≥S2) was seen in in 24% CHB individuals.

Among CHB individuals, 5/53 were HBeAg positive. There was one HBsAg positive individual, a 9-year-old male, born in the U.S. who was anti-HBs positive, and both HBeAg and anti-HBe negative with normal liver tests and undetectable HBV DNA. Among the 5 HBeAg positive patients, median HBV DNA was 8374 (IQR 144–343545) IU/mL and median ALT 39 (IQR 32–66) U/L. Three CHB individuals were anti-HCV reactive, and only one individual had detectable HCV RNA.

**Figure 1.**
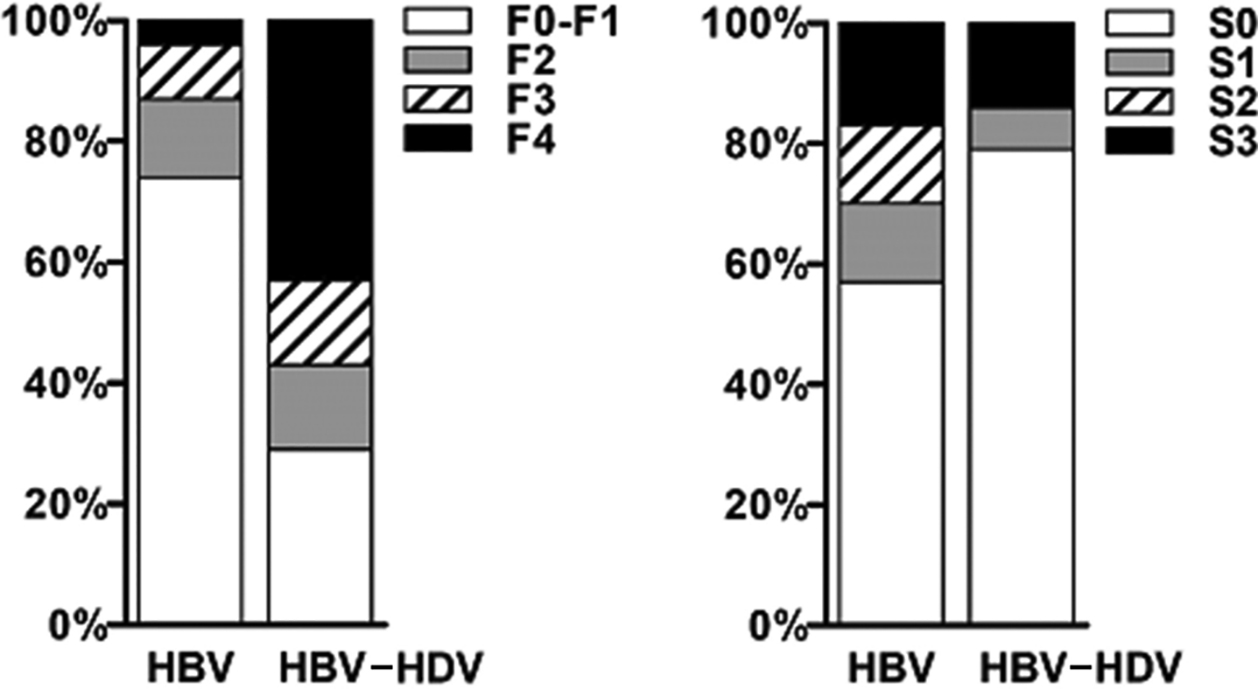
Assessment of liver fibrosis and steatosis in HBV mono- vs HBV/HDV super-infected patients. HBV mono- (n=23) and HBV/HDV super-infected (n=14) patients underwent the assessment of the degree of liver fibrosis (left) and steatosis (right) via controlled transient elastography (FibroScan®, Echosens, Paris, France) and controlled attenuation parameter (CAP®, EchoSens, Paris, France), respectively. HBV/HDV super-infected patients had more fibrosis compared to HBV mono-infected patients (p=0.01052). There was no difference in steatosis grades (p=NS). F0-F1: none to mild fibrosis; F2: moderate fibrosis; F3: severe fibrosis; F4: cirrhosis. S0: no steatosis; S1: mild steatosis; S2: moderate steatosis; S3: severe steatosis.

HBV genotype that could be evaluated in 23 CHB individuals who had sufficient quantity of HBV-DNA titer for the nested-PCR followed by the Sanger sequencing was almost exclusively D1 (n=20) or A1 (n=3) genotypes. Among the 34 individuals with HBV mono-infection, 5 met criteria for anti-viral therapy (15). Delta hepatitis antibodies were detected in 21/53 (39.6%) CHB individuals and 18/53 (34.0%) were HDV-RNA positive. Concordance of anti-HDV and HDV RNA testing was 83%. Individuals with HDV RNA positive were older compared to HDV RNA negative individuals (41 ± 10 vs 34 ± 10 years old, respectively, p=0.025). The youngest individuals who were HDV RNA or HDV antibody positive were 33 and 28 years old, respectively. Compared to HDV negative individuals, HDV RNA positive individuals had significantly higher ALT levels, lower HBV DNA levels, higher Fibrosis-4 scores and greater liver stiffness (Table 1). The prevalence of advanced fibrosis (≥ F3) by FibroScan was significantly higher in HDV RNA positive individuals compared to HDV RNA negative patients (57% vs. 13%, p=0.013) (Figure 1).

### Prevalence and Clinical Characteristics of HCV Infection

Forty-eight individuals (9.1%) were anti-HCV positive and 19 (3.6%) were HCV RNA positive (Table 2). Those with CHC were older than those with CHB or super-infected HBV-HDV (p<0.01). The mean age of CHC individuals was 45 (range 25–81) years. The prevalence of anti-HCV positive was higher among those born between 1945–1965 compared to those born after 1965 (18.8% vs 7.9%, p=0.025), but there was no difference in prevalence of HCV RNA positive among the birth cohorts (4.2% vs. 3.3%, p=0.764). Genotype 1b was predominant. Among the 13 individuals with BMI measurement, median BMI was 25.71 (range 19.2–31.9) kg/m^2^. FibroScan was performed on 26 individuals and mean liver stiffness was 7.3 (range 3.4–23.3) kPa and 15% had advanced fibrosis. Significant steatosis by CAP (≥S2) was seen in 31% CHC individuals.

## DISCUSSION

This was the single most comprehensive study of chronic viral hepatitis among Mongols in which demographic, virologic and clinical characteristics were evaluated. Our study confirms that chronic viral hepatitis B, D and C infections are highly prevalent among Mongols (3–5,8,16–22). Since universal vaccination was implemented in Mongolia in 1991, prevalence rate of CHB has decreased but rates vary between rural versus urban areas, gender and age of individuals. In a study performed in 2004 of 1145 Mongolian children aged 7–12 year old, overall HBsAg positivity rate was 5.2% (23). In our study, the prevalence rate of CHB was 9.9% which is expected since most of our tested subjects were born after 1991. The prevalence of CHC in this study was 9.1%. These prevalence rates for CHB and CHC in this study are comparable to a study of Mongols in the Washington D.C. (9). Rates of CHB and CHC among Mongols are among the highest among Asian-Americans where disaggregated data are available (24,25).

Despite a younger cohort of CHB individuals, the proportion of HBeAg positive individuals was very low, even among Mongols under the age of 30. Only one individual had an immune tolerant profile. Hepatitis B genotypes D and A were prevalent in this study. The distribution of HBV genotypes is similar to European and Mediterranean countries in contrast to other Asian-Americans with CHB where genotypes B and C predominate (26,27). Interestingly, these Western countries with predominant genotypes B and D were part of the Mongol Empire at the height of its expansion during the 13^th^ and 14^th^ centuries (28). Indeed, not only does the pattern of HBV genotype distribution compare, but the HDV prevalence rate among Mongols resembles that of Central Asia and Turkey (5).

In contrast to other Asians with CHB, HDV was highly prevalent among this cohort of US-living Mongols and comparable to prevalence rates reported in Mongolia (2–7, 10, 20–22). In this study, there were no HDV infected individuals under 30 years of age (among 93 individuals screened), suggesting that HDV among Mongols maybe an acquired risk behavior in adults. The use of unsterile instruments in the setting of dental work, acupuncture and folk remedies such as blood-letting are thought to be potential sources of HBV/HDV infection (17,19,21). Clinical characteristics of chronic viral hepatitis among Mongols have not been previously described. Our study showed that Mongols with HBV-HDV super- infection were more likely to have more advanced liver disease with more severe liver fibrosis, lower level of HBV DNA and higher ALT levels.

The prevalence of HDV among CHB patients in the U.S. is approximately 3% in various settings and as high as 8% among CHB patients in the San Francisco area, most of whom were non-Hispanic Caucasians (29–32) and intravenous drug users (33). Historically, in Los Angeles during the 1980s, HDV infection was noted among predominantly among individuals who inject drugs and in men who have sex with men with CHB (34,35). A significant number of HDV infected individuals were co-infected with HIV (36). The demographics of CHB infection in Los Angeles have changed significantly, and the majority of CHB patients currently seen are Asian and Hispanic with no reported high-risk behaviors (37) and a low prevalence of HDV infection (unpublished data) although there may be sampling bias (29,30).

Mean age of Mongols with CHC was significantly older than HBV and HBV-HDV individuals suggesting that exposure to HCV occurs in adults. Our study found an HCV infection rate of 9.7% among U.S.-living Mongols, which is consistent with reports of other Mongolian cohorts (3,4,17,18). Only about one third of anti-HCV positive individuals were viremic. Unfortunately, treatment history was not captured in the questionnaire so some of these individuals may have been successfully treated or spontaneously cleared HCV. The prevalence of HCV in Mongolia is significantly higher than neighboring China, but in line with prevalence rates of other central Asian countries such as Uzbeskistan (11.3%) and Turkmenistan (5.6%) (38,39). HCV genotype 1b was predominant which is similar to other Asian countries (25,38,39).

Our study also found that obesity and non-alcoholic fatty liver (NAFLD) were highly prevalent among Mongols with CHB or CHC. The age-standardized prevalence of obesity in men and women in Mongolia between 2005 and 2013 increased from 10.8% to 17.6% and from 18.9% to 26.4%, respectively. Using Asian-specific BMI cutoff values for men and women, the age-standardized prevalence of obesity between 2005 and 2013 increased from 20.0% to 32.8% and 33.4% to 43.7%, respectively (40). There is a growing interest in the interaction of chronic viral hepatitis and the natural history of NAFLD as the rate of obesity rises in Asia. Concomitant NAFLD and chronic viral hepatitis have been shown to cause more severe liver disease (41,42) although studies from Korea and China show CHB was associated with a reduced risk of NAFLD (43, 44).

There were limitations to this study. The number of individuals was not large, but this represents one of the largest cohorts of Mongols studied and the most comprehensive evaluation. Not all patients had BMI measurement and FibroScan since only patients who tested positive for either HBsAg or anti-HCV were called back. Information on risk factors and anti-viral therapy was not collected.

In summary, this study provides important epidemiological data of viral hepatitis in a high- risk ethnic group, Mongols living in the US, are at high risk for CHB and CHC infections. Chronic delta hepatitis infection was present in one third of CHB individuals with nearly half showing advanced fibrosis. It is critical to screen all CHB Mongols for HDV and provide education and appropriate medical care to prevent super-infection of HDV in CHB individuals. This study also illustrates the success of a partnership of community advocacy group and medical program in recruiting at-risk individuals for screening, education and providing linkage to medical care.

## Figures and Tables

**Table 1.** Comparison of Mongolian Patients with Chronic Hepatitis B mono-infection Versus Co-infection with Hepatitis D Virus.

	HBV mono-infection (N=35, 66%)	HBV-HDV co-infection (N=18, 34%)	p-value
Age (years)	34 ± 10	41 ± 10	**0.025**
Sex (male)	57%	61%	1
BMI (kg/m^2^)	26.5 ± 5.6	24.9 ± 5.5	0.63
Years in US (years)	6.1 ± 4.5	6.2 ± 6.0	0.959
Hepatitis B e Antigen	9%	11%	1
AST (U/L)	25 (22–30)	57 (40–113)	**<0.001**
ALT (U/L)	29 (18–37)	71 (44–91)	**<0.001**
Albumin (g/dL)	4.9 (4.6–5.1)	4.8 (4.4–4.8)	0.075
Total bilirubin (mg/dL)	0.5 (0.4–0.7)	0.7 (0.6–1.0)	**0.007**
Platelet count (× 10^9^ per L)	237 ± 86	173 ± 44	**0.001**
AFP (ng/mL)	3 (2–7)	6 (3–10)	0.126
HBV DNA (IU/mL)	212(24–4653)	0 (0–131)	**0.011**
Liver stiffness (kPa)	6.1 ± 2.4	11.1 ± 5.6	**0.007**
FibroScan Fibrosis score F3-F4	13%	57%	**0.013**
FIB-4 Score	0.69 (0.57–0.93)	1.68 (1.36–2.79)	**<0.001**

Values provided as mean ± standard deviation, median (interquartile range), or column percentage.

Comparisons between groups were made by student’s t-test, Mann-Whitney U test, or chi-square analysis, as appropriate.

**Table 2.** Characteristics of Mongolian Patients with Chronic Hepatitis C (N=48)

	HCV
Age (years)	45 ± 12
Sex (male)	40%
Years in US (years)	7.1 ± 6.1
AST (U/L)	26 (20–41)
ALT (U/L)	26 (18–42)
Albumin (g/dL)	4.8 (4.6–5.0)
Total bilirubin (mg/dL)	0.5 (0.4–0.6)
Platelet count (× 10^9^ per L)^≠^	229 ± 53
AFP (ng/mL)	3 (2–4)
Liver stiffness (kPa)*	7.3 ± 3.8
FibroScan Fibrosis score F3-F4*	15%
CAP score*	233 ± 58
FibroScan Steatosis score ≥ S2	31%
FIB-4 Score^≠^	1.01 (0.74–1.34)

Values provided as mean ± standard deviation, median (interquartile range), or column percentage.

Comparisons between groups were made by student’s t-test, Mann-Whitney U test, or chi-square analysis, as appropriate.

* 26 of 38 patients with available data

≠ 46 of 48 patients with available data
